# Aflatoxin exposure assessed by aflatoxin albumin adduct biomarker in populations from six African countries: REVIEW ARTICLE

**DOI:** 10.3920/WMJ2017.2284

**Published:** 2018-08-01

**Authors:** Y. Xu, Y.Y. Gong, M.N. Routledge

**Affiliations:** 1 School of Medicine, University of Leeds, Leeds LS2 9JT, United Kingdom; 2 School of Food Science & Nutrition, University of Leeds, Leeds LS2 9JT, United Kingdom

**Keywords:** mycotoxin, sub-Saharan Africa, geographical variation

## Abstract

Aflatoxins are a group of carcinogenic mycotoxins that have been implicated to have other adverse health impacts, including child growth impairment and immune function suppression. Aflatoxin B_1_ is the most toxic and most common of the aflatoxins. Contamination of various food crops is common in sub-Saharan Africa, particularly in staple crops such as maize and groundnuts, leading to chronic dietary exposure in many populations. For many years we have used the aflatoxin albumin adduct as a biomarker of aflatoxin exposure, assessed using a competitive inhibition enzyme linked immunosorbent assay (ELISA). Here, we review our recent studies of human exposure in six African countries; Gambia, Guinea, Kenya, Senegal, Tanzania and Uganda. This data shows the widespread exposure of vulnerable populations to aflatoxin. Geometric mean (95% confidence interval) levels of the biomarker ranged from 9.7 pg/mg (8.2, 11.5) in Ugandan children to 578.5 pg/mg (461.4, 717.6) in Kenyan adolescents during an acute aflatoxicosis outbreak year. We describe how various factors may have influenced the variation in aflatoxin exposure in our studies. Together, these studies highlight the urgent need for measures to reduce the burden of aflatoxin exposure in sub-Saharan Africa.

## 1. Introduction

Aflatoxins are a group of naturally occurring mycotoxins produced by various strains of *Aspergillus* spp., and are prevalent in crops in Africa and south Asia area. As secondary metabolites, it is generally considered that mycotoxins such as aflatoxin confer some advantage to the fungi producing them, and as production is often triggered by environmental conditions such as drought, it is probable that this involves competition with other microorganisms when resources are scarce (Magan and Aldred, 2007). There are four types of aflatoxin (B_1_, B_2_, G_1_ and G_2_), which may be produced in different quantities by different species. Aflatoxin B_1_ (AFB_1_) is the most potent type of aflatoxin and has been classified as a group one carcinogen by IARC, as have naturally occurring mixtures of aflatoxin (IARC, 2002).

Here we will review our recent papers reporting aflatoxin biomarker levels in six African countries, with some additional context of crop contamination reports and the impact of aflatoxin exposure in sub-Saharan Africa.

## 2. Health impact of aflatoxin exposure

Due to the frequent, and often high, contamination of staple foods such as maize and groundnuts by aflatoxin, populations of many African countries are at risk of chronic exposure, which can frequently reach very high levels. Acute high exposure leads to outbreaks of aflatoxicosis, presenting as nausea, vomiting, abdominal pain and fever leading to liver failure that is potentially fatal. The most severe recorded outbreak was in Kenya in 2004, during which 125 deaths and hundreds of cases of acute hepatic failure occurred (Azziz-Baumgartner *et al.*, 2005; Strosnider *et al.*, 2006). Recently, an outbreak in Tanzania resulted in at least 14 deaths (Buguzi, 2016; Kamala *et al.,* 2018). The chronic exposure to lower levels of aflatoxin in the diet has been established as a causative agent contributing to primary hepatocellular carcinoma (IARC, 2002; Ross *et al.*, 1992; Wogan, 1992), with co-exposure to hepatitis B virus (HBV) enhancing the carcinogenicity of the aflatoxin based on the evidence from China and East Asia (Qian *et al.*, 1994, Sun *et al.*, 2002). In affected regions of China, previous high levels of primary liver cancer have been greatly reduced following national HBV immunisation programmes and a shift in staple food based on maize to the less susceptible rice (Sun *et al.*, 2013).

Aflatoxin exposure has also been associated with child growth impairment (Castelino *et al.*, 2015; Gong *et al.*, 2004), hepatomegaly (Gong *et al.*, 2012) and immune function suppression (Jiang *et al.*, 2005, 2008; Turner *et al.*, 2003). These associations raise extremely important questions about the contribution of aflatoxin to the high prevalence of child malnutrition in Africa and add to the need for effective interventions to reduce aflatoxin exposure to be developed (IARC, 2015).

## 3. Contamination of crops

The fungi that produce aflatoxin are widespread throughout sub-Saharan Africa with warm and humid conditions being favourable for fungal growth. In the field, environmental stress such as drought can promote fungal growth on crops and production of the toxin. Aflatoxin production is also promoted during storage by warm, humid conditions, so incomplete drying of crops is a factor in increased accumulation of aflatoxin during storage. Levels of contamination may vary from year to year, season to season and as a result of factors such as local farming and storage practices, local soil conditions and local climate.

Consuming contaminated foods is the primary way for people to be exposed to aflatoxin. There are different types of aflatoxin that are detected in foods, but AFB_1_ is the most toxic. Table 1 shows some examples of levels of aflatoxin contamination in crops and foods from studies carried out in five of the six African countries, in which we have recently assessed aflatoxin exposure by biomarker analysis (no aflatoxin crop data was available for Guinea). Such surveys highlight the widespread occurrence of aflatoxin in food crops in sub-Saharan Africa and also show the variation in aflatoxin contamination by year and location. For example, Daniel *et al.* (2011) assessed aflatoxin contamination levels in maize samples collected from hundreds of local households in Kenya from 2005 to 2007, and found significantly higher aflatoxin levels in 2005 and 2006 compared to 2007 (geometric mean (GM) 12.9 and 26.0 vs 1.95 μg/kg, *P*<0.001). Kaaya and Kyamuhangire (2006) determined the variation of aflatoxin contamination levels in maize samples collected from three agroecological zones in Uganda. Mean levels were highest in the midaltitude moist climate and lowest in the dry highlands, which reflect the contribution of moist conditions to aflatoxin production during storage of crops. They also found higher level of aflatoxin in maize stored more than six months (30.2 μg/kg) compared to samples stored less than six months (20.5 μg/kg). The range of contamination seen in Uganda was lower than that seen in Kenya. In addition to the environmental conditions variation, aflatoxin contamination was also found higher in groundnuts than in maize in Uganda and Gambia. The very wide range of levels reported in some studies highlight the heterogeneous nature of aflatoxin contamination. Exposure is also, of course, influenced by consumption levels which can also vary widely from location to location. For example, the Food and Agricultural Organisation of the United Nations (FAOSTAT) report maize consumption of 171 g/person/day in Kenya, 128 g/person/day in Tanzania, 62 g/person/day in Senegal and 52 g/person/day in Uganda (Ranum *et al.*, 2014). In some countries, such as the Gambia, groundnuts are consumed more frequently than maize and will therefore contribute more to aflatoxin exposure.

**Table 1:** Levels of aflatoxin contamination reported in maize, maize based foods and/or groundnuts in five African countries.

Country	Crop/foodstuff (sample size)	Total aflatoxin (or AFB_1_)^1^ level, range (μg/kg)	Aflatoxin level, GM (95% CI)^2^, median or mean ± SD as indicated (μg/kg)	Reference
Kenya	Market maize (n=350)	1-46,400	20.5 (13.4-31.4)	Lewis *et al.*, 2005
	Households maize			Daniel *et al.*, 2011
	2005 (n=298)	0.11-48,000	12.9	
	2006 (n=165)	0.30-24,400	26.0	
	2007 (n=253)	<LOD-2,500	2.0	
	Total (n=716)	<LOD-48,000	9.1	
	Maize kernel (n=20)	18-480	53	Kilonzo *et al.*, 2014
Tanzania	Household maize (n=120)	5-90^c^	38^a^	Kimanya *et al.,* 2008
	Mixed maize (n=4)	1.0-120.0	1.3	Manjula *et al.*, 2009
	Maize based flour	0.5-364^c^	1.2^a^	Kimanya *et al.*, 2014
	Maize (n=60)	2-1,081	65	Kamala *et al.*, 2015
	Feed samples (n=37)	<LOD-2.0	0.4	Mohammed *et al.*, 2016
	Maize porridge samples (n=101)			Geary *et al.*, 2016
	Nyabula	0.2-27.6	4.5	
	Kikelelwa	0.2-34.5	5.8	
	Kigwa	0.2-25.8	4.7	
Uganda	Maize kernels			Kaaya *et al.*, 2006
	Mid-altitude (moist) (n=80)	0-32	20.5	
	Mid-altitude (dry) (n=80)	0-22	18.0	
	Highland (n=80)	0-15	12.4	
	Foods (n=100)	0-55	15.7	Kitya *et al.*, 2010
	Markets groundnuts (n=33)	0-540	103.1±36.6^b^	Baluka *et al.*, 2017
		0-849^c^	180.7±51^b^	
Senegal	Groundnuts (n=20)	0.55-15.33	4.43±2.13^b^	Diedhiou *et al.*, 2012
Gambia	Groundnuts (n=18)	18-943	162^a^	Hudson *et al.*, 1992
	Maize (n=9)	2-35	9.7^a^	

^1^ Superscript (c) denotes aflatoxin B_1_ level; LOD limit of detection.

^2^ GM = geometric mean; 95% CI = 95% confidence interval; superscript (a) denotes median value; superscript (b) arithmetic mean value ± standard deviation (SD).

## 4. Biomarkers of exposure

Exposure to aflatoxin may be estimated by measuring contamination levels in food, together with recording food consumption. However, the heterogeneous nature of food contamination means that it can be difficult to get accurate estimates of individual exposure. For this, biomarkers of aflatoxin exposure in body fluids give more useful information. Biomarkers of AFB_1_ exposure are the products of AFB_1_ metabolism and include urinary AFB-N^7^-guanine adducts (AFB-N^7^-Gua), aflatoxin M_1_, aflatoxin Q_1_, and aflatoxin P_1_ (Groopman *et al.*, 1993, 1994; Ross *et al.*, 1992; Wang *et al.*, 1996) and aflatoxin albumin adducts (AF-alb) in blood (Sabbioni *et al.,* 1990). Biomarkers in urine can only reflect the recent (24 h) aflatoxin exposure (Groopman *et al.*, 1992; Zhu *et al.*, 1987), while AF-alb in serum integrates exposure over the preceding two to three months (Skipper and Tannenbaum, 1990; Wild *et al.*, 1992). The application of biomarker methods for studying exposure provides a more direct measurement of the exposure of individuals and populations and facilitates studies into the health risks associated with aflatoxin exposure (Routledge and Gong, 2011). The potential health impacts of aflatoxin, usually determined through use of biomarker measurements of exposure, has been recently reviewed (Gong *et al.*, 2016).

In our studies of aflatoxin exposure in African populations, a competitive inhibition ELISA with a limit of detection of 3 pg/mg albumin was used to measure AF-alb in serum (Chapot and Wild, 1991). In this method, human serum samples were processed to extract albumin, then 2 mg albumin was subjected to pronase digestion and C18 cartridge clean up to purify the adduct. Positive and negative quality controls were analysed along with each batch of samples. Although there is potential for the antibody used in the ELISA to cross-react with other aflatoxins, the fact that AFB_1_ is most likely to give rise to AF-alb and that the albumin has been purified before analysis means that the adducts measured most likely represent AFB_1_ exposure, although this cannot be definitively confirmed. This method has been validated against aflatoxin intake in adults and children (Routledge *et al.*, 2014; Wild *et al.*, 1992).

## 5. Aflatoxin-albumin levels in populations of six African countries

In recent years we have conducted a series of research projects on aflatoxin exposure and child health, utilising the ELISA technique to analyse blood samples from several African populations (see Table 2).

### Geographical variation

**Figure 1:**
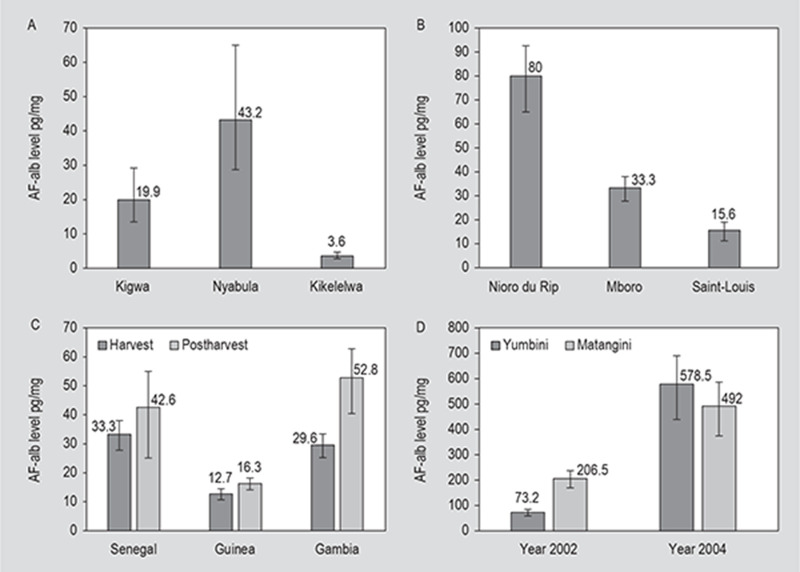
Variation in aflatoxin-albumin geometric mean (GM) values in different populations. (A) AF-alb GM in three agro-ecological zones in Tanzania (Shirima *et al.*, 2013). (B) AF-alb GM in three agro-ecological zones in Senegal (Watson *et al.*, 2015). (C) Effect of post-harvest storage on AF-alb GM in Senegal, Guinea and Gambia (Castelino *et al.*, 2014; Watson *et al.*, 2015, 2016). (D) Large variation in AF-alb GM in two neighbouring Kenyan villages in 2002 and 2004, a year of an aflatoxicosis outbreak(Gong *et al.*, 2012). Error bars show 95% confidence intervals.

In Table 2, GM levels and 95% CI of AF-alb in samples from populations studied in Tanzania, Gambia, Senegal, Uganda, Kenya and Guinea are summarised. The results from these studies show variation in exposure in populations from different countries. In these studies, mean levels of AF-alb were observed to be highest in Kenya (Gong *et al.*, 2012) and lowest in Uganda (Asiki *et al.*, 2014), with exposure seen in all populations tested. Whether this represents consistent geographical differences or is partly due to year to year variation in toxin levels is not clear. It is important to recognise that although the biomarker does integrate exposure over the previous two to three months, these results represent biomarker levels at a particular point in time and from particular populations within the countries in question. However, in some studies measurements in the same year in populations from different regions do show significant variations. This pattern of geographical variation was seen in Tanzania, where samples were collected from children in three locations in geographically distant regions of Tanzania-Nyabula in Iringa region, Kigwa in Tabora region and Kikelelwa in Kilimanjaro region (Shirima *et al.*, 2013) (Figure 1A). In this study serum samples were taken at baseline when children were 6-14 months old and then again at six and twelve months later. At six and twelve month time points, the AF-alb levels were lowest in children from Kikelelwa, which is in a more elevated and dryer region than the other two sites and hence crops are less susceptible to aflatoxin contamination during storage. AF-alb levels were highest in samples from Kigwa on the third visit, with mean levels of 48.8 pg/mg. Geographical variation in exposure was also observed in Senegal with AF-alb levels significantly higher in a population in Nioro du Rip, South Senegal (GM = 80 pg/mg) compared to one from Saint-Louis in North-West Senegal (GM = 15.6 pg/mg) (*P*<0.001), with intermediate levels (GM = 33.3 pg/mg) in a population in Mboro in Western Senegal (Watson *et al.*, 2015) (Figure 1B). These values were measured in samples taken at harvest time. Samples taken three to four months later after a period of groundnut storage showed increased levels of exposure in Saint-Louis and Mboro, consistent with seasonal variation seen elsewhere, but not in Nioro du Rip, where there was a fall in GM levels to 58.6 pg/mg, which may reflect reduced consumption of groundnuts in this period.

**Table 2:** Levels of aflatoxin-albumin adduct in six studies in sub-Saharan Africa.

Country	Participants	Aflatoxin-albumin level, GM (95% CI)^1^ or range as indicated pg/mg	References
Uganda	100 adults	11.5 (10.2, 13.0)	Asiki *et al.*, 2014
	96 children (<3 years old)	9.7 (8.2 , 11.5)	
Kenya	(2002)		Gong *et al.*, 2012
	124 children from Yumbini	73.2 (61.6, 87.0)	
	94 children from Matangini (6-17 years old) (2004)	206.5 (175.5, 243.0)	
	124 children from Yumbini	578.5 (466.4, 717.6)	
	94 children from Matangini (6-17 years old)	492.0 (397.3, 609.2)	
Gambia	134 pregnant women (18-45 years old)	early pregnancy 34.5 (29.3, 40.7) later pregnancy 41.8 (34.7, 50.3)	Castelino *et al.*, 2014
Senegal	168 adults (39±12 years old)	total 45.7 (range 5.5-588.2) harvest vs postharvest Nioro du Rip: 80.0 vs 58.6 Mboro: 33.3 vs 42.6 Saint-Louis: 15.6 vs 25.6	Watson *et al.*, 2015
Tanzania	166 children (6-14 months)	recruitment 4.7 (3.9, 5.6) 6 months after recruitment 12.9 (9.9, 16.7) 12 months after recruitment 23.5 (19.9, 27.7)	Shirima *et al.*, 2015
Guinea	305 children (28.8±8.4 months)	harvest 12.7 (10.9, 14.7) postharvest 16.3 (14.4, 18.5)	Watson *et al.*, 2016

^1^ GM = geometric mean; CI = confidence interval.

In the Kenya study (Gong *et al.*, 2012) differences were seen in two populations of schoolchildren from adjacent communities. In samples from children in the Matangini school, the mean levels of AF-alb in 2002 were markedly higher (206.5 pg/mg) than those seen in Yumbuni (73.2 pg/mg) even though these villages are close together (Figure 1D). The difference between exposure levels between children from two schools in the same area in 2002 reflects the different local conditions. Yumbuni is in a more elevated, dryer location, whereas Matangini is located in a lower altitude with several streams providing greater humidity. The more humid atmosphere in Matangini would promote aflatoxin production on crops during storage compared to Yumbuni, showing that local microclimates can influence risk of exposure.

### Seasonal variation

An increase in aflatoxin in crops during storage is common, and we have observed higher levels of AF-alb in serum samples taken post-harvest compared to at harvest time, for example in Senegal (Watson *et al.*, 2015) and in Guinea (Watson *et al.*, 2016) (Figure 1C). In Gambia, where groundnuts are the main source of aflatoxin exposure, we have measured AF-alb levels in pregnant women (Castelino *et al.*, 2014). The women were recruited over a 12 month period, as part of the ENID trial, a nutrient supplementation trial (Moore *et al.*, 2012), so that some blood samples were taken during the rainy season and some during the dry season. We saw that in pregnant women the mean level of AF-alb varied with season, being higher in the dry season (60.8 pg/mg) than in the rainy season (28.1 pg/mg). The dry season (October to May) is the post-harvest period and this difference in seasonality may reflect higher levels of aflatoxin in stored nuts as well as a reduction in other foods as the dry season progresses. We also found higher mean levels of AF-alb in samples taken in late versus early pregnancy, although this was only significant in the dry season. It has been suggested that this could reflect higher food intake during later pregnancy.

As well as seasonal variations in exposure, aflatoxin contamination of crops, and hence exposure levels, can vary year on year. Serum samples taken from children in a Yumbuni school in the Makueni District of Kenya in 2002 and again in 2004 revealed a large difference in AF-alb levels, with a mean of 73.2 pg/mg in 2002 and a dramatically high mean of 578.5 pg/mg in 2004 (Gong *et al.*, 2012) (Figure 1D). These exceptionally high levels of AF-alb reflected high levels of aflatoxin contamination in 2004, a year in which there was a serious outbreak of acute aflatoxicosis in this region (Azziz-Baumgartner *et al.*, 2005).

### Diet and age

Groundnuts and maize are not the only crops susceptible to aflatoxin contamination but tend to be the main contributory staple crops. In the Senegal study (Watson *et al.,* 2015) as well as taking blood samples for AF-alb measurement, total aflatoxin level in groundnuts and maize samples were measured and food frequency data were recorded. Participants who consumed groundnuts/maize more than four times per week were considered to be in the high groundnuts/maize consumption group, with others belonging to the low consumption group. Significantly high levels of AF-alb were determined in people of high groundnuts consumption (62.8 pg/mg) compared to those of low consumption (24.0 pg/mg) (*P*<0.001). Notably, nearly all of the people tested in Nioro du Rip at harvest time were in the high groundnut consumption group, which helps to explain the high levels of AF-alb in serum from this population as aflatoxin levels were found to be higher in groundnuts compared to maize. This sort of information may be useful as supporting evidence to underpin arguments for changes in dietary habits as a means of reducing aflatoxin exposure, although it is recognised that such changes will require major policy shift with large resource implications. In a recent pilot study in Gambia, we showed that education to follow a simple hand-sorting intervention to remove mouldy nuts prior to food preparation could dramatically reduce aflatoxin contamination (Xu *et al.*, 2017).

In Guinea, the potential impact of aflatoxin exposure on micronutrient levels was assessed by measuring serum concentrations of vitamin A, vitamin E, β-carotene and zinc in addition to AF-alb (Watson *et al.*, 2016). It was found that children in the highest aflatoxin exposure quartile were more likely to be vitamin A and zinc deficient compared to children in the lowest quartile of aflatoxin exposure. This association highlights the potential for aflatoxin exposure to interact with important markers of nutrition.

In children, breastfeeding is protective against aflatoxin exposure. Although AFM_1_ is present in breast milk of exposed mothers, this is less toxic than AFB_1_ and in breastfed children AF-alb are lower than in weaned children (Gong *et al.*, 2004). In our Uganda study, children less than 3 years old who were exclusively breastfed had less than half of AF-alb levels than those eating food supplements (Asiki *et al.*, 2014). Likewise in Tanzania, Shirima *et al.* (2013) reported a positive correlation between AF-alb level and maize intake in children. Fully weaned children had about twofold higher levels of AF-alb compared to those who were partially weaned (24.7 vs 10.7 pg/mg, respectively). In this population the GM of AF-alb also showed an upward trend with age (Shirima *et al.*, 2015). The GM level of AF-alb was 6.1 pg/mg in children under 16 months, 16.2 pg/mg among 16-18 months old children and up to 19.8 pg/mg in children over 18 months. This reflects increased exposure with increased family food intake and age related differences in older children are not apparent. No significant age related difference in AF-alb levels were seen among children aged 6-17 years old in Kenya (Gong *et al.*, 2012). All of the studies found that there is no significant difference of aflatoxin exposure according to gender.

Aflatoxin exposure *in utero* can also impact on child growth, as seen in an earlier study in which higher AF-alb in maternal blood being a predictor of both low birth weight and infant height gain (Turner *et al.*, 2007). In the Gambia, we found AF-alb levels in pregnancy to be associated with differences in white blood cell DNA methylation in the children at six months of age, suggesting a possible mechanism by which exposure to aflatoxin *in utero* can influence outcomes in children such as growth (Hernandez-Vargas *et al.*, 2015).

## 6. Intervention to prevent aflatoxin exposure

Aflatoxin contamination in European countries are controlled by legislation, and most of the European countries reported no or very little aflatoxin in grains (Streit *et al.*, 2012). In contrast, sub-Saharan Africa is very susceptible to food safety issues due to poorer economy, subsistence agriculture, food insecurity and lack of regulation enforcement. The climate in sub-Saharan Africa provides a suitable temperature and humidity for fungal growth, therefore, aflatoxin contamination can happen during crops growth, post-harvest and storage. A number of intervention methods for reducing aflatoxin levels have been described. Rachaputi *et al.* (2002) found that early harvesting and threshing can reduce the aflatoxin contamination of groundnuts. Using fertilization and improved agriculture irrigation have been shown to control fungi growth, but the high costs are a barrier to implementation in Africa (Khlangwiset and Wu, 2010). Therefore, simple practices during harvesting and storage are more feasible. Proper drying of groundnuts and maize to low moisture (less than 10%) before storage can significantly reduce aflatoxin (Awuah and Ellis, 2002; Turner *et al.*, 2005). Other practices, such as hand-sorting, washing, and roasting before eating have been shown to be effective and acceptable to local populations (Wagacha and Muthomi, 2008; Xu *et al.*, 2017). There is significant investment ongoing to introduce atoxigenic strains of aflatoxin (tradename Aflasafe) into sub-Saharan Africa, which has shown great potential for reducing contamination in field trials (Bandyopadhyay *et al.*, 2016).

## 7. Conclusions

The recent studies reviewed here have highlighted that aflatoxin exposure is widespread in sub-Saharan Africa, adding to the evidence from previous studies. It is common to see geographical variation in exposure levels, which can often be explained by local climate, storage practices, dietary intake and socioeconomic conditions. There is often seasonal variation that is related to increased aflatoxin accumulation during crop storage, and there can be large variation between years. Once weaning has started, children are quickly exposed to levels as high as adults (or higher when expressed as intake/kg/body weight), so breastfeeding, which is important for good early nutrition, is also important to protect against early exposure to aflatoxin. There is a clear and urgent need for interventions to reduce aflatoxin exposure across sub-Saharan Africa and elsewhere.
